# Florid Cementoosseous Dysplasia: A Rare Case Report

**DOI:** 10.1155/2013/946583

**Published:** 2013-09-03

**Authors:** Mehmet Fatih Şentürk, Recep Kestane, Elif Naz Yakar, Ahmet Keskin

**Affiliations:** ^1^Department of Oral and Maxillofacial Surgery, Faculty of Dentistry, University of Süleyman Demirel, East Campus, 32260 Çünür Isparta, Turkey; ^2^Department of Oral and Maxillofacial Surgery, Faculty of Dentistry, University of Ankara, Ankara, Turkey; ^3^Department of Dentomaxillofacial Radiology, Faculty of Dentistry, University of Ankara, Ankara, Turkey

## Abstract

Florid cementoosseous dysplasia (FCOD) is a rare, benign, fibroosseous, and multifocal dysplastic lesion of the jaw that consists of cellular fibrous connective tissue with bone and cementum-like tissue. FCOD is most commonly found in middle-aged black women, is generally asymptomatic, and is usually detected during radiological examination. FCOD associated with multiple impacted teeth and bone expansion is a very rare phenomenon, and there are only a few familial cases reported in the literature. In this report, a 35-year-old male Turkish patient is presented who was diagnosed with nonfamilial FCOD from clinical, radiological, and histopathological findings. To our knowledge this is the first case of the nonfamilial FCOD with this many impacted teeth and severely expanded bones.

## 1. Introduction

Florid cementoosseous dysplasia (FCOD) is a benign, fibroosseous, and multifocal dysplastic lesion of the jaw that consists of cellular fibrous connective tissue with bone and cementum-like tissue [1]. FCOD was previously known as gigantiform cementoma, multiple cementoossifying fibroma, sclerosing osteitis, multiple enostosis, and sclerotic cemental masses of the jaws. It was first comprehensively described by Melrose et al. [2]. This lesion is most commonly found in middle-aged black women, although it also may occur in Caucasians and Asians [3, 4]. The etiology of FCOD is unknown, and there is no clear explanation for its gender and racial predilections [5]. Clinically these lesions are often asymptomatic. Symptoms such as dull pain or drainage are almost always associated with exposure of the sclerotic calcified masses in the oral cavity [2, 4, 5]. Radiographically, the lesions appear as multiple sclerotic masses located in two or more quadrants, usually in the tooth-bearing regions. They are often confined within the alveolar bone [6]. 

A search of the literature showed that only a few cases have been reported concerning the familial form of FCOD associated with multiple impacted teeth. However, no examples were found of the nonfamilial form of FCOD associated with multiple impacted teeth. In this study, a very rare case of nonfamilial FCOD associated with multiple impacted teeth and bone expansion is presented and the current literature regarding this lesion reviewed.

## 2. Case Presentation

**Figure 1:**
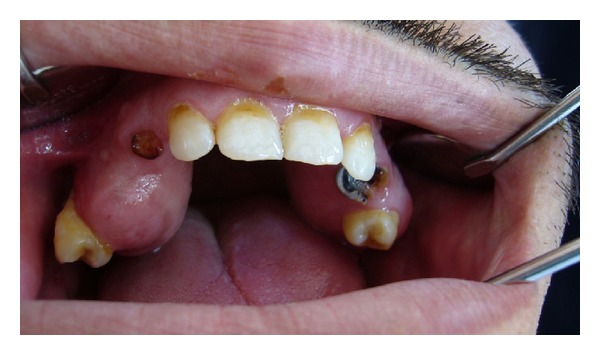
Preoperative view of the maxilla with bone expansions intraorally.

**Figure 2:**
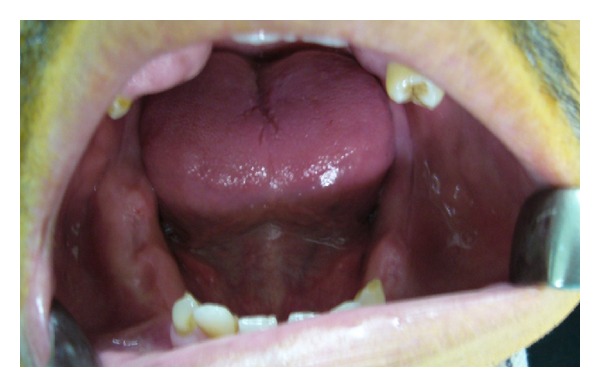
Preoperative mandible intraorally.

**Figure 3:**
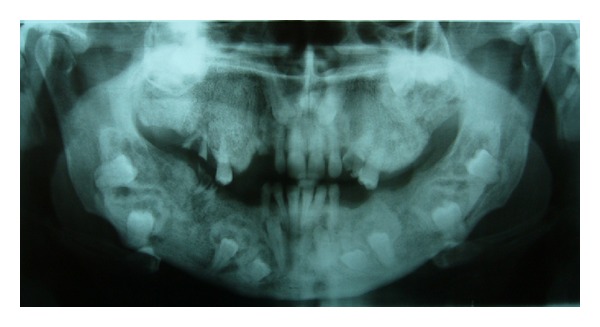
Preoperative panoramic radiograph showing multiple impacted teeth and bone expansion.

**Figure 4:**
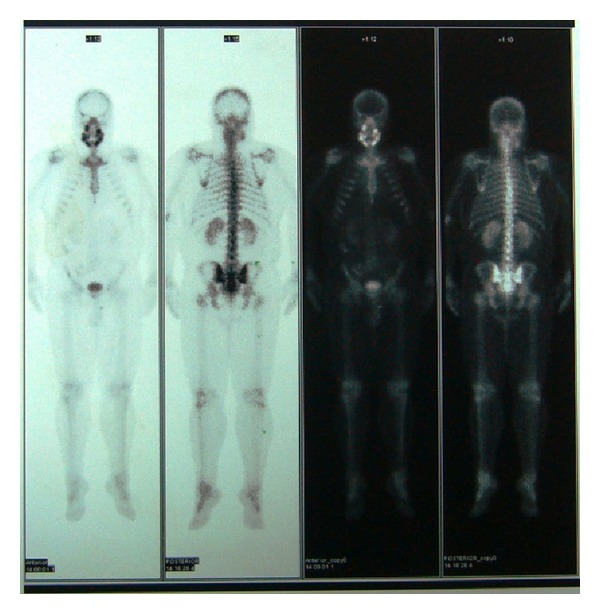
Scintigraphic bone scans showing no increased osteoblastic activity on the other bony formations.

**Figure 5:**
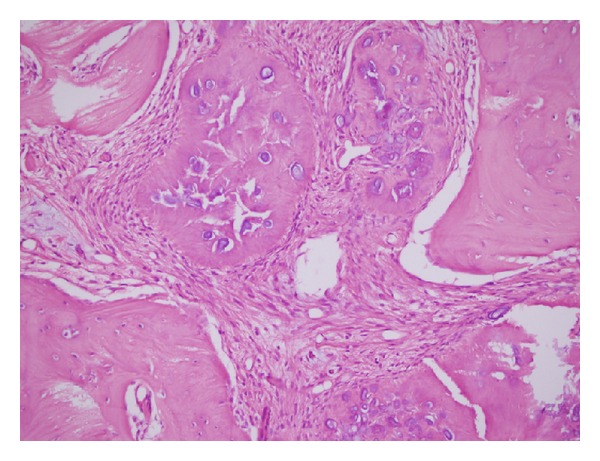
Histopathologically rounded cement bone-like structures showing irregular lamellation are seen in fibrous stroma consisting of fibroblastic cells (HEx100).

**Figure 6:**
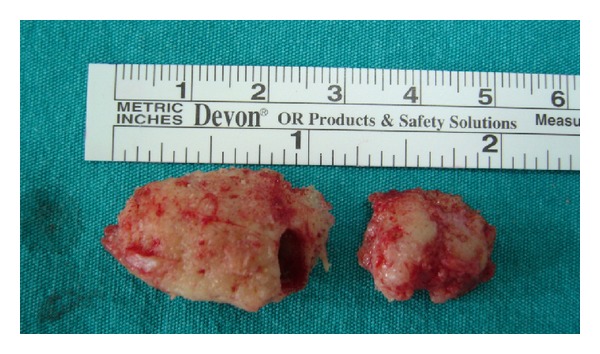
Bony segments removed from maxilla.

**Figure 7:**
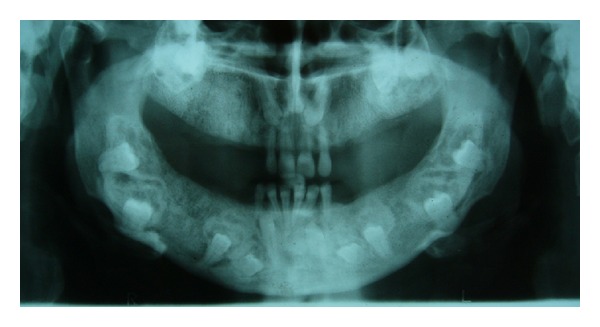
Postoperative panoramic radiograph.

**Figure 8:**
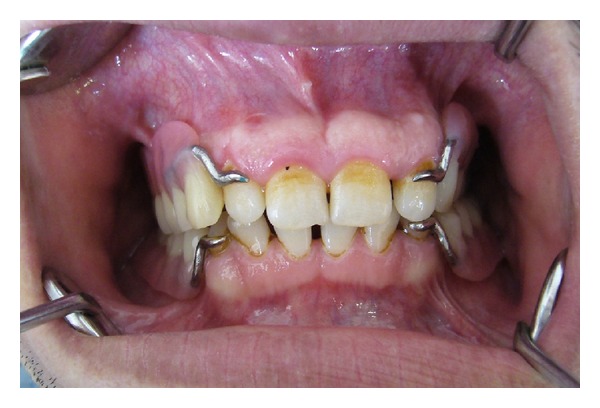
Postoperative 1-year view of occlusion with removable partial prosthesis.

**Figure 9:**
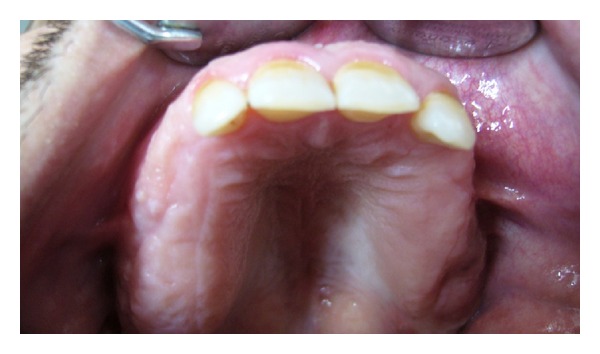
Postoperative 1-year view of maxillary arch intraorally.

**Figure 10:**
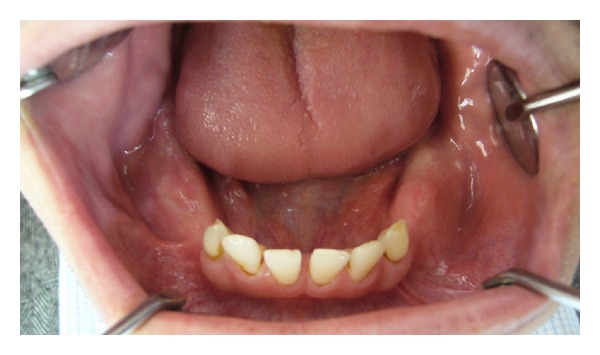
Postoperative 1-year view of mandibular arch intraorally.

A 35-year-old male patient was referred to the Department of Oral and Maxillofacial Surgery, Faculty of Dentistry, at Ankara University, with severe swelling, notably in the maxilla. The patient had no systemic symptoms. Clinical examination revealed expansion of the bone and partially edentulous areas on both the maxilla and mandible. Several decayed roots were discovered in both jaws, and some of the erupted teeth were malposed due to bone expansion. The overlying gingiva and mucosa were normal with no clinical signs of inflammation; the patient stated that he had never experienced pain in any part of his jaws (Figures 1 and 2). The familial history was taken and some of the family members were examined, but no familial aspects of the disease could be established. Radiological examination revealed multiple, diffuse, lobular radioopacities throughout the edentulous areas of the maxilla and mandible with multiple impacted teeth. Most of the impacted teeth seemed to be pushed through the periphery of the jaws by the expansile lesions (Figure 3). The serum alkaline phosphatase level was within normal limits, and a scintigraphic bone scan showed no increased osteoblastic activity on the other bony formations (Figure 4). Thus, the differential diagnosis of FCOD was made rather than Paget's disease or Gardner's syndrome. However, the case was extraordinary in many ways and surgery was required to allow prosthetic rehabilitation. A bone biopsy was performed to support the initial diagnosis. Histopathologically, rounded cement bone-like structures, showing irregular lamellation, were seen in fibrous stroma consisting of fibroblastic cells (Figure 5). When clinical, radiological, and histopathological findings were evaluated together, the definitive diagnosis was made of FCOD. Cortical bone expansions were recontoured in two operations under local anesthesia. No impacted teeth were extracted due to their location. The remaining roots and malposed teeth were extracted, and the removed bony segments were sent for histopathological examination (Figure 6). Histopathological findings confirmed the diagnosis of FCOD once again. The postoperative course was uneventful. A partial, removable prosthesis was fabricated 1 month after the surgery. Care was taken to avoid any trauma as a result of the prosthesis. Routine controls were performed every 6 months. The postoperative 1-year course was uneventful (Figures 7, 8, 9, and 10). The patient has been followed up for 16 months with no complication. Followup is continuing.

## 3. Discussion

FCOD refers to a set of radiolucent-radiopaque periapical and interradicular lesions involving the mandible bilaterally and sometimes the maxilla. It is basically an extended form of periapical cementoosseous dysplasia. These lesions are also asymptomatic dysmorphic bone-cementum complexes. Radiographs show large, radiolucent, mixed, or most often, dense radiopaque masses, limited to the periapical alveolar bone. They do not involve the inferior border, except through direct focal extension, and do not occur in the rami [7]. The present case was a severe form of FCOD, involving all four quadrants, including the angle and the basal bone in some areas of the mandible.

FCOD typically occurs in middle-aged black women [8]. Melrose et al. [2] reported a study with 34 cases of similar lesions, of which 32 were black women (in a predominantly Caucasian population) with a mean age of 42. The present case of a 35-year-old Turkish man may represent the first of such a rare combination of features being reported in the English and Turkish language literature.

FCOD should be differentiated from Paget's disease, chronic diffuse osteomyelitis, and Gardner's syndrome. FCOD has no other skeletal changes, skin tumors, or dental anomalies. Thus FCOD can be differentiated from Gardner's syndrome. Paget's disease is polyostotic and shows raised alkaline phosphatase level which is not a consistent feature of FCOD. Chronic diffuse sclerosing osteomyelitis is not confined to tooth-bearing areas. It is a primary inflammatory condition of the mandible, with cyclic episodes of unilateral pain and swelling. The affected lesion of the mandible exhibits a diffuse opacity with poorly defined borders [9].

FCODs are most often painless and detected through routine radiographs [10, 11]. Their presence is not usually associated with expansion, but rare cases may show mild expansion [7]. FCOD affecting multiple family members appears to be quite uncommon. There are only a few reports in which the hereditary nature of the lesion could be demonstrated [12–15]. Unlike the sporadic cases, the familial form is characterized by more expansile lesions, which may recur after surgery, and it tends to occur in younger subjects. In all of the familial cases reported, FCOD appears to be inherited as an autosomal dominant trait with variable phenotypic expression [12, 14–16]. Toffanin et al. [17] reported a case of FCOD affecting multiple family members. Some of the affected subjects had multiple impacted teeth, and one also had marked expansion of the symphyseal region. That patient underwent resection and reconstruction of the mandibular body with a free osteomyocutaneous fibula graft. In the present case, no familial aspects of the disease could be established. The case was also painless, but it showed several impacted teeth and marked expansion in both jaws. The nonfamilial form of FCOD very rarely shows such a combination.

Multiple impacted teeth are a rare phenomenon in cases diagnosed as FCOD. A search of the literature showed only two reports of FCOD associated with multiple impacted teeth. Toffanin et al. [17] reported 4 cases of FCOD with multiple impacted teeth; 12 were the highest number of impacted teeth among those cases. Srivastava et al. [18] also reported a case of FCOD associated with multiple impacted teeth. Seventeen impacted teeth were recorded in this case. In both of these reports, the FCOD was familial in nature. However, in our case 15 impacted teeth were seen, and there were no familial aspects. To our knowledge, our case has the maximum number of impacted teeth among nonfamilial FCOD cases reported thus far. 

Normally, the diagnosis of FCOD in the jaws is made through the clinical and radiographic features [19]. In the asymptomatic patient, it is probably wise to keep the patient under observation without surgical intervention. A biopsy is not required to confirm the diagnosis as this is usually established radiographically. It is not normally justified to surgically remove these lesions, as the surgery involved can be extensive. Instead, followup and recontouring are recommended when cortical expansion occurs [20]. Whenever surgical treatment is planned, the lack of vascularity of the lesion and increased risk of osteomyelitis should be considered. The affected area undergoes changes from normal vascular bone into an avascular cementum-like lesion. Furthermore, complete removal of necrotic tissue may result in a large discontinuity defect [21]. However, in lesions causing pain and disturbance, surgery and the risks it entails might be necessary for adequate treatment. However, recontouring should be the treatment of choice where there is only cortical expansion and mucosal perforation due to the cementoosseous lesions [22]. Our case had cortical expansion in all four quadrants making prosthetic rehabilitation impossible. As a result we decided to perform recontouring surgery to obtain adequate interocclusal space for prosthesis. We performed a bone biopsy conservatively to confirm the initial diagnosis of FCOD. Histologic examination supported our clinical and radiological diagnosis. Subsequently, we performed surgery in all four quadrants in two sessions under local anesthesia.

In conclusion, bone expansion and multiple impacted teeth are very rarely seen in nonfamilial forms of FCOD. If the lesions and impacted teeth are asymptomatic, it is wise to avoid surgical intervention. However, if the expansion makes prosthetic rehabilitation impossible in edentulous jaws, recontouring surgery may be the best choice to obtain sufficient space for prosthesis.

## References

[1] Wakasa T, Kawai N, Aiga H, Kishi K (2002). Management of florid cemento-osseous dysplasia of the mandible producing solitary bone cyst: report of a case. *Journal of Oral and Maxillofacial Surgery*.

[2] Melrose RJ, Abrams AM, Mills BG (1976). Florid osseous dysplasia. A clinical pathologic study of thirty four cases. *Oral Surgery Oral Medicine and Oral Pathology*.

[3] Miyake M, Nagahata S (1999). Florid cemento-osseous dysplasia report of a case. *International Journal of Oral and Maxillofacial Surgery*.

[4] Waldron CA (1985). Fibro-osseous lesions of the jaws. *Journal of Oral and Maxillofacial Surgery*.

[5] Beylouni I, Farge P, Mazoyer JF, Coudert JL (1998). Florid cemento-osseous dysplasia: report of a case documented with computed tomography and 3D imaging. *Oral Surgery, Oral Medicine, Oral Pathology, Oral Radiology, and Endodontics*.

[6] Damm DD, Fantasia JE (2001). Multifocal mixed radiolucencies. Florid cemento-osseous dysplasia. *General Dentistry*.

[7] Marx RE, Stern D (2004). *Oral and Maxillofacial Pathology: A Rationale for Diagnosis and Treatment*.

[8] Ong S-T, Siar C-H (1997). Florid cemento-osseous dysplasia in a young Chinese man. Case report. *Australian Dental Journal*.

[9] Kim JH, Song BC, Kim SH, Park YS (2011). Clinical, radiographic and histological findings of florid cemento-osseous dysplasia: a case report. *Imaging Science in Dentistry*.

[10] Minhas G, Hodge T, Gill DS (2008). Orthodontic treatment and cemento-osseous dysplasia: a case report. *Journal of Orthodontics*.

[11] Said-al-Naief NA, Surwillo E (1999). Florid osseous dysplasia of the mandible: report of a case. *Compendium of Continuing Education in Dentistry*.

[12] Young SK, Markowitz NR, Sullivan S, Seale TW, Hirschi R (1989). Familial gigantiform cementoma: classification and presentation of a large pedigree. *Oral Surgery Oral Medicine and Oral Pathology*.

[13] Sedano HO, Kuba R, Gorlin RJ (1982). Autosomal dominant cemental dysplasia. *Oral Surgery Oral Medicine and Oral Pathology*.

[14] Coleman H, Altini M, Kieser J, Nissenbaum M (1996). Familial florid cemento-osseous dysplasia–a case report and review of the literature. *The Journal of the Dental Association of South Africa*.

[15] Musella AE, Slater LJ (1989). Familial florid osseous dysplasia: a case report. *Journal of Oral and Maxillofacial Surgery*.

[16] Cannon JS, Keller EE, Dahlin DC (1980). Gigantiform cementoma: report of two cases (mother and son). *Journal of Oral Surgery*.

[17] Toffanin A, Benetti R, Manconi R (2000). Familial florid cemento-osseous dysplasia: a case report. *Journal of Oral and Maxillofacial Surgery*.

[18] Srivastava A, Agarwal R, Soni R, Sachan A, Shivakumar GC, Chaturvedi TP (2012). Familial florid cemento-osseous dysplasia: a rare manifestation in an Indian family. *Case Reports in Dentistry*.

[19] Sarmento DJDS, de Brito Monteiro BV, de Medeiros AMC, da Silveira EJD (2013). Severe florid cemento-osseous dysplasia: a case report treated conservatively and literature review. *Oral and Maxillofacial Surgery*.

[20] Jerjes W, Banu B, Swinson B, Hopper C (2005). Florid cemento-osseous dysplasia in a young Indian woman. A case report. *British Dental Journal*.

[21] Bencharit S, Schardt-Sacco D, Zuniga JR, Minsley GE (2003). Surgical and prosthodontic rehabilitation for a patient with aggressive florid cemento-osseous dysplasia: a clinical report. *Journal of Prosthetic Dentistry*.

[22] Kayaaltı Özarslan S, Yılmaz HH, Aksoy MÇ, Baykul T (2011). Florid cemento-osseous dysplasia: a case report. *Journal of Dental Faculty of Atatürk University*.

